# 2′-*O*-Galloylhyperin Isolated From *Pyrola incarnata* Fisch. Attenuates LPS-Induced Inflammatory Response by Activation of SIRT1/Nrf2 and Inhibition of the NF-κB Pathways *in Vitro* and *Vivo*

**DOI:** 10.3389/fphar.2018.00679

**Published:** 2018-06-27

**Authors:** Peng Wang, Chang Gao, Na Guo, Sun-Dong Zhang, Wei Wang, Li-Ping Yao, Jing Zhang, Thomas Efferth, Yu-Jie Fu

**Affiliations:** ^1^Key Laboratory of Forest Plant Ecology, Ministry of Education, Northeast Forestry University, Harbin, China; ^2^Department of Obstetrics and Gynecology, Peking University Third Hospital, Beijing, China; ^3^Institute of Pharmacy and Biochemistry, Johannes Gutenberg University, Mainz, Germany; ^4^Beijing Advanced Innovation Center for Tree Breeding by Molecular Design, Beijing Forestry University, Beijing, China

**Keywords:** NF-κB, SIRT1, anti-inflammation, 2′-*O*-galloylhyperin, Nrf2

## Abstract

2′-*O*-galloylhyperin, a major compound of *Pyrola incarnata* Fisch., possesses a variety of biological and pharmacological activities, including anti-oxidative and anti-inflammatory activities. Nevertheless, the underlying molecular mechanisms of 2*′-O*-GH in microbial infection and sepsis are not clear. In this study, we investigated the anti-inflammatory effects of 2*′-O*-GH. We found that 2*′-O*-GH significantly reduced the production of TNF-α, IL-6, and nitric oxide (NO), suppressed the expression levels of iNOS, blocked the translocation of NF-κB from the cytosol to nucleus, and decreased the MAPK activation in LPS-activated RAW 264.7 cells. 2*′-O*-GH also enhanced the nuclear translocation of Nrf2 and up-regulated the expression of heme oxygenase-1 (HO-1) and SIRT1. In addition, the administration of 2*′-O*-GH attenuated the TNF-α and IL-6 production in the serum, infiltration of inflammatory cells, liver tissue damage, and the mortality rate of LPS-challenged mice. Moreover, 2*′-O*-GH significantly upregulated Nrf2 and SIRT1 expression and inhibited the inflammatory responses in the liver of septic mice. The collective data indicate that 2*′-O*-GH could potentially be a novel functional food candidate in the treatment of sepsis.

## Introduction

Inflammation has been recognized as a complex physiological process that eliminates injurious stimuli and initiates the healing process regulated by the immune defensive system. However, excessive inflammation is also involved in many human diseases including arthritis, metabolic syndrome, cancer, and atherosclerosis (Laveti et al., 2013). LPS, a complex glycolipid in the outer membrane of Gram-negative bacteria, can innate immune system, because it binds the CD14/TLR4/MD2 receptor complex in many cell types, especially in monocytes and macrophages, which modulates cytokine networks by producing pro-inflammatory cytokines such as TNF-α, IL-6, and pro-inflammatory mediators including nitric oxide (NO) (Mo et al., 2014; Wu et al., 2017). In addition, LPS also stimulates the production of ROS *via* the activation of NADPH-oxidase in macrophages (Menon et al., 2014). ROS, as modulators of redox-sensitive signaling molecules, are involved in the onset of immune responses by mediating transcription factor as well as signaling pathway activation, such as NF-κB and MAPK pathways (Li et al., 2018). Moreover, HO-1, tightly regulated by Nrf2, inhibits the secretion of pro-inflammatory mediators through the inactivation of NF-κB in LPS-stimulated RAW 264.7 cells (Wang et al., 2016). Therefore, the reduction of inflammatory mediators and ROS in macrophages provides a useful therapeutic approach against various inflammatory diseases.

SIRT1, as a member of highly conserved NAD^+^-dependent class III histone deacetylases, plays an important role in the regulation of cellular oxidative stress response, metabolism, senescence as well as apoptosis (Hwang et al., 2013). The knockdown of SIRT1 in mouse RAW264.7 macrophages significantly increased LPS-stimulated expression of pro-inflammatory cytokines such as TNF-α, IL-6, and IL-1β, mediated by enhanced NF-κB activity (Zhang et al., 2017). Similarly, up-regulation of SIRT1 decreased IL-6 and TNF-α expression induced by cigarette smoke extract in endothelial cells (Zhang et al., 2008). In addition, resveratrol (a SIRT1 agonist) treatment suppressed the over-expression of pro-inflammatory molecules and decreased acute lung injury in a sepsis mouse model induced by LPS *via* activation of SIRT1 and elimination of the oxidative stress (Chiavaroli et al., 2010; Li et al., 2013). All these data indicate that SIRT1 exhibits pronounced anti-inflammatory properties, and pharmacological activation of SIRT1 may be a potential therapeutic strategy for inflammation-related diseases.

Functional foods (also called nutraceuticals, pharmafoods, and designer foods) are made from natural ingredients and offer, in addition to their nutritional value, a specific health advantage due to the preventive, protective, and/or curative activity *in vivo* as a supplemented food additive (Dullius et al., 2018). *Pyrola* [*P. incarnata* Fisch.], a member of the *Pyrolaceae* family, is an edible herb that is widely applied in food and medical industry (Yao et al., 2015). In China, *Pyrola*, known as *lushoucha* and *changleyuye*, has been used as tea to enhance immunity and slow the aging process (Sun et al., 2011). In addition, as a traditional Chinese medical herb, it is used to treat rheumatoid arthritis and gastric or pulmonary hemorrhage (Luo et al., 2004). Among the bioactive compounds of *Pyrola*, 2*′-O*-GH is a major polyphenolic compound with strong antioxidant activity (Yao et al., 2015). In our previous study, we mainly focused on its significant anti-oxidant properties, including superoxide radical scavenging activity and DPPH radical scavenging activity (Yao et al., 2013; Wang et al., 2017). However, the key pharmacological efficacies of 2′-*O*-GH against inflammation and redox balance have not been reported yet. In addition, SIRT1 plays a role in mediating the inflammatory response, and its relationship to 2′-*O*-GH during sepsis has not been studied. Therefore, we investigated whether 2′-*O*-GH treatment could attenuate the LPS-induced inflammatory response and redox imbalance in RAW264.7 macrophages (*in vitro*) and LPS-challenged mice (*in vivo*) by regulating SIRT1 expression.

## Materials and Methods

### Reagents and Antibodies

2*′-O*-GH (purity ≥ 98%) was isolated from *Pyrola* [*P. incarnata* Fisch.], and the chemical structure was identified in our laboratory (Yao et al., 2013, 2015). A 10-mM stock solution of 2*′-O*-GH was prepared in DMSO and stored at -80°C.

Polyclonal antibodies against JNK, phospho-JNK (Thr183/Tyr185), p38 MAPK, phospho-p38 MAPK (Thr180/Tyr182), p42/p44 MAPK, phospho-p42/44 MAPK (Thr202/Tyr204), phosphor-IKKα/β (Ser176/177), NF-κB p65, SIRT1, β-actin and inhibitor compounds U0126 (a ERK inhibitor), SB203580 (p38 MAPK inhibitor), and SP600125 (JNK inhibitor) were purchased from Beyotime Institute of Biotechnology (Beijing, China). DMSO, LPS (from *Escherichia coli O111: B4*), NAC and MTT were obtained from Sigma (St. Louis, MO, United States). DEX was supplied by Xi’an Lijun Pharmaceutical Company Limited (Xi’an, China). Other reagents and chemicals were purchased from Beijing Chemical Reagents Co. (Beijing, China). Secondary antibodies were obtained from Beyotime Institute of Biotechnology (Beijing, China). Deionized water was purified by a Milli Q Water Purification system from Millipore (Millipore Corp., Bedford, MA). PVDF membrane was purchased from Millipore (Millipore Corp., Bedford, MA).

### Cell Culture

RAW264.7 macrophage cells, purchased from the CBCAS (Cell Bank of the Chinese Academic of Sciences, Shanghai, China), were cultured in DMEM (Invitrogen) containing 10% (v/v) FBS (Hyclone) and antibiotics (100 U/ml penicillin and 100 μg/mL streptomycin) (Hyclone) at 37°C in a humidified 5% CO_2_ incubator.

### Cell Viability Assay

RAW264.7 cells were seeded into 96-well plates at a density of 2 × 10^4^ cells per well 24 h before treatment. Cells were treated with various concentrations of 2′-*O*-GH for 2 h, and followed with or without LPS 100 ng/mL for 24 h. Viabilities were determined using MTT assays, as described previously (Wang et al., 2016).

### Measurement of NO and Cytokine

RAW264.7 cells were plated into 24-well plates for 24 h before pretreated with or without different concentrations of 2′-*O*-GH for 2 h, then were stimulated with or without LPS (100 ng/mL) for 24 h. The production of TNF-α and IL-6 in the culture supernatant were determined by using enzyme-linked immunosorbent assay kits according to the manufacturer’s instructions. Nitric oxide was measured by the detection of its stable oxidative metabolite, nitrite. In brief, 100 μL of the culture media were mixed with 50 μL of Griess reagents I and II for 10 min at room temperature. Absorbance was measured in a microplate reader at 540 nm. The amount of nitrite in the samples was evaluated based on the sodium nitrite standard curve.

### Nuclear, Cytoplasmic Extraction, and Western Blotting

The extraction and isolation of nuclear and cytoplasmic protein were performed according to the Nuclear and Cytoplasmic Protein Extraction Kit (Beyotime, China). First, cells were centrifuged for 5 min at 1200 rpm at 4°C, and the pellet was dissolved with cytoplasmic protein extraction agent A supplemented with PMSF. After vortex for 5 s, the tubes were incubated for 10–15 min on ice to promote lysis. Next, add the cytoplasmic protein extraction agent B, vortex for 5 s and incubated on ice for 5 s. Then the samples were centrifuged for 5 min at 14,000 *g* at 4°C, and the supernatant, consisting of the cytosolic fraction, was immediately frozen for further analysis. The pellet was resuspended in nuclear protein extraction agent supplemented with PMSF. After vortexing the tubes 15–20 times for 30 min and centrifuging for 10 min at 14,000 g, the supernatants containing the nuclear extracts were obtained. Subsequently, protein concentration was determined using a BCA protein assay kit according to the protocol from the manufacturer. Equal amount of total protein and nuclear protein were separated by 10% SDS-PAGE followed by electroblotted to PVDF membrane (Millipore, MA, United States). Then the membrane was blocked with 1% bovine serum albumin for 2 h at room temperature. Afterwards, the membrane was incubated with primary antibodies (1:500) and subsequently with secondary antibodies (1:1000). The abundance of target proteins was densitometrically determined using Quantity Ones (Bio-Rad Laboratories, Berkeley, CA, United States) and was expressed as fold changes after normalization to the invariant control.

### Transfection and Luciferase Assay

RAW 264.7 cells were co-transfected with pNF-κB-Luc plasmid and pRL-TK vector (Promega, Madison, WI) using Lipofectamine 3000 reagent (Invitrogen Corp., Carlsbad, CA) according to the manufacturer’s instruction. After 24 h of transfection, cells were pretreated with 2′-*O*-GH for 2 h and then stimulated with LPS (100 ng/mL) for another 12 h. Cells were lysed and luciferase activities were performed using the dual-luciferase reporter assay kit (Promega).

### Detection of ROS

The intracellular accumulation of ROS was monitored using the fluorescent probe DCFH-DA. Briefly, after treatment, cells were collected and washed twice with PBS, then PBS were removed, and 10 μM of DCFH-DA was added and incubated for 30 min at 37°C in the dark. The fluorescence was measured by flow cytometry and fluorescence microscopy. The fold-increase of ROS generation was compared with the vehicle-treated cells, which were arbitrarily considered as onefold.

### Immunofluorescence and Confocal Microscopy

RAW 264.7 cells were cultured on sterile cover-slips in six-well plates. Then the cells were pretreated with 2′-*O*-GH 2 h prior to LPS (100 ng/mL) stimulation. After treatment, cells were fixed with 4% paraformaldehyde for 10 min and washed with PBS for three times. Following this, cells were permeabilized with 0.2% Triton-100 for 20 min and incubated with a blocking buffer for 1 h to suppress non-specific binding. Next, cells were incubated with the primary NF-κB p65 antibody overnight at 4°C, followed by incubation with the Alexa Fluor 488-conjugated secondary antibody for 1 h. For nuclear staining, the cells were then counterstained with DAPI for 5 min before observation. Images were captured using a fluorescence microscopy.

### Animals and Experimental Sepsis

Male ICR mice (25–30 g) were obtained from Liaoning Changsheng Biotechnology Co. Ltd. (SCXK(Liao)-2015-0001). The animals were allowed free access to food and water. They were maintained in a controlled environment at a temperature of 22 ± 1°C and 50 ± 5% relative humidity with 12 h light/dark cycles and acclimatized for at least 1 week before use. The animal experiments were approved by the Laboratory Animal Center of Northeast Forestry University and were performed in accordance with the guidelines of the U.S. National Institutes of Health. For septic mortality experiment, mice were intraperitoneally injected with 2′-*O*-GH 10 or 50 mg/kg for 12 and 1 h before LPS injection (30 mg/kg, i.p.). Mice were monitored every 12 h for 72 h. Serum and liver tissues were collected for 12 h after LPS injection.

### Histopathology and Biochemistry Analysis

The harvested livers were fixed in 4% paraformalclehyde, dehydrated with an ethanol, and embedded in paraffin then cut into 4 μm sections. The sections were stained with H&E. Pathological changes were evaluated under a light microscope. All specimens were histologically assessed by two experienced pathologists, and the extent of histological changes was scored according to a modified Ishak system (Cheng et al., 2018). Levels of TNF-α and IL-6 in serum were measured using corresponding assay kits according to the manufacturer’s instructions. The biochemical parameters, including AST, ALT in serum, SOD and GSH in liver tissues, were measured with enzymatic assays, according to the manufacturer’s instructions (Nanjing Jiancheng Bioengineering Institute, Nanjing, China).

### Reverse Transcriptase Polymerase Chain Reaction

Total RNA was isolated with Trizol reagent according to the manufacturer’s instruction. Total RNA (1.0 μg) was converted to cDNA using PrimeScript^TM^ RT Reagent Kit with gDNA Eraser (Takara, Dalian, China). The cDNA was amplified by with following primer sequences: SIRT1 forward and reverse primers: ATGACAGAACGTCACACGCC and AACAATCTGCCACAGCGTCA (1 min of 94°C denaturation, 30 s of 60°C annealing, and 1 min 72°C extension); Actin forward and reverse primers: GTGCTATGTTGCTCTAGACTTCG and ATGCCACAGGATTCCATACC (1 min of 94°C denaturation, 30 s of 60°C annealing, and 1 min 72°C extension). Following amplification, PCR products were electrophoresed on 2% agarose gel.

### Statistical Analysis

All results were expressed as mean values ± standard deviation (*n* = 3). Differences between groups were calculated by one-way analysis of variance (ANOVA) followed by Tukey *post hoc* multiple comparison tests. All statistics were calculated using the STATISTICA program (StatSoft, Tulsa, OK). Correlations were calculated using the ReglinP function and inverted Student’s *t* test. *p* < 0.05 was considered as statistically significant.

## Results

### 2′-O-GH Suppressed the Production of IL-6, IL-1β, TNF-α, NO, and Expression of iNOS Protein in LPS-Stimulated RAW264.7 Cells

**FIGURE 1:**
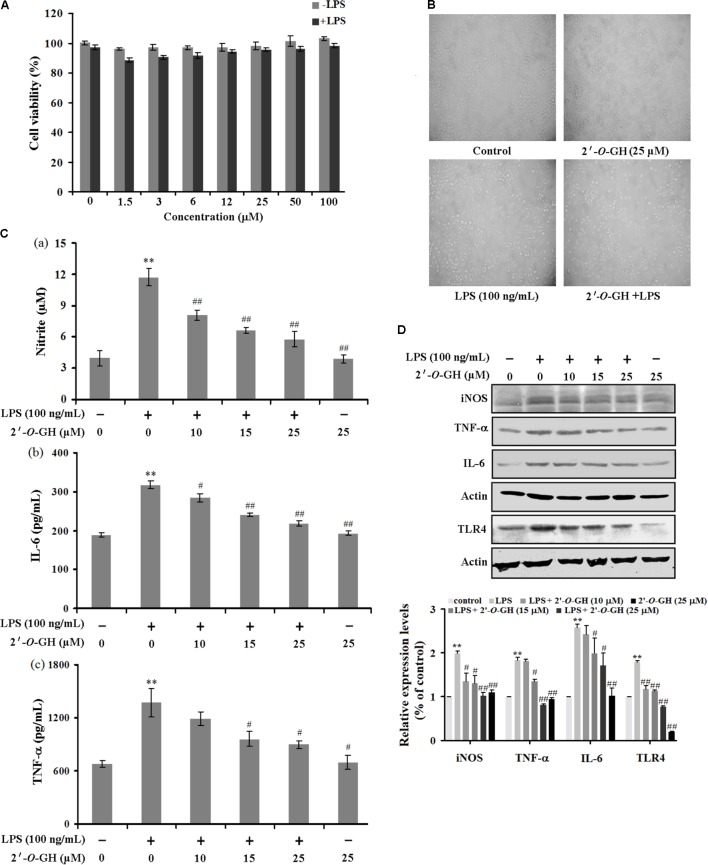
Effects of 2′-*O*-GH on LPS-stimulated pro-inflammatory gene expression. RAW264.7 cells were pretreated with 2′-*O*-GH for 2 h or left untreated and then stimulated with LPS (100 ng/mL) for 24 h. **(A)** Cell viability was measured by the MTT assay. **(B)** Representative images of RAW 264.7 cells treated with 2′-*O*-GH and LPS (100 × magnification). **(C)** The concentrations of nitrite, TNF-α, and IL-6 were measured in the culture medium by Griess reagent or ELISA kits. **(D)** Cells were pretreated with different concentrations of 2′-*O*-GH for 2 h. LPS (100 ng/mL) was then incubated for 18 h. Whole-cell lysates were subjected to SDS-PAGE, and the protein levels of iNOS, TNF-α, IL-6, and TLR4 were determined by immunoblot analysis. β-actin was used as control. Quantitative data were presented as mean ± SEM (*n* = 3). ^∗∗^*p* < 0.01 versus control, ^#^*p* < 0.05, ^##^*p* < 0.01 versus LPS (one-way ANOVA followed by Tukey *post hoc* multiple comparison tests).

**FIGURE 2:**
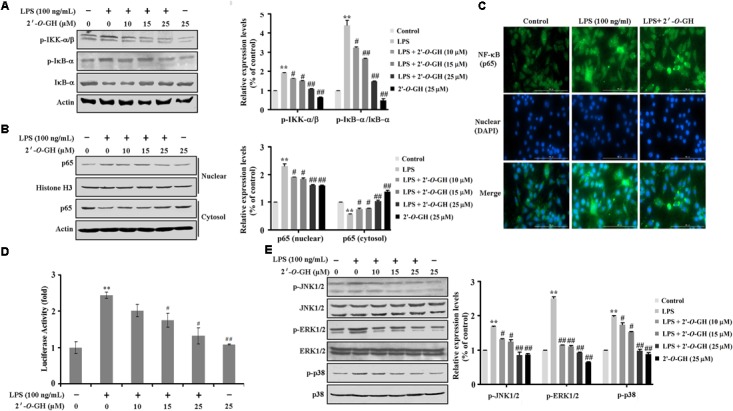
Effects of 2′-*O*-GH on LPS-induced activation of NF-κB and MAPKs pathway in RAW 264.7 macrophages cells. RAW264.7 cells were pretreated with 2′-*O*-GH for 2 h or left untreated and then with/without LPS (100 ng/mL) for 15 min **(A)**, 1 h **(B–D)** or 30 min **(E)**. **(A)** Lysates were analyzed by Western blot using antibodies against p-IκB-α, IκB-α, and p-IKKα/β. **(B)** Nuclear and cytosol extracts were subjected to Western blot analysis to determine the level of p65. Histone H3 was used as nuclear marker and β-actin as cytosol protein marker for standardization. **(C)** The translocation of p65 to the nucleus was analyzed by confocal microscopy. Cells were immunostained using FITC for p65 and DAPI for nucleus. **(D)** Cells were transfected with a pNF-κB-luc reporter vector and the pRL-TK vector as internal control. After 24 h of transfection, cells were treated with 2′-*O*-GH for 2 h and then stimulated with LPS (100 ng/mL) for 12 h. Cells were then harvested, and luciferase activity levels were determined as described in the Experimental Section. **(E)** Whole cell lysates were analyzed by Western blot using antibodies against activated MAPKs. Quantitative data were presented as mean ± SEM (*n* = 3). ^∗∗^*p* < 0.01 versus control, ^#^*p* < 0.05, ^##^*p* < 0.01 versus LPS (one-way ANOVA followed by Tukey *post hoc* multiple comparison tests).

First of all, we determined whether 2′-*O*-GH affected cell viability using MTT assay. Cells were treated with different concentrations of 2′-*O*-GH for 24 h, and the percentage of cell viability in RAW 264.7 macrophages were shown in **Figures 1A,B**, 2′-*O*-GH did not have significant cytotoxicity to RAW 264.7 cells in the absence or presence of LPS (100 ng/mL). We also investigated the production of the NO and cytokines (TNF-α and IL-6) in LPS-stimulated RAW 264.7 cells. As seen in **Figures 1C,D**, pretreatment of 2′-*O*-GH attenuated LPS-induced release of these pro-inflammatory molecules, including NO, TNF-α, and IL-6, in a concentration-dependent manner, which was consistent with Western blot analysis. To investigate whether toll-like receptors were involved in the effect of 2′-*O*-GH on LPS-induced activation, protein expression of TLRs were observed after LPS treatment. The results showed that TLR4 but not TLR2 protein expression was up-regulated significantly in LPS-treated cells compared with untreated cells. However, 2′-*O*-GH blocked the LPS-induced increasing of TLR4 (**Figure 1D**).

### 2′-O-GH Suppressed LPS-Induced NF-κB and MAPK Activation in RAW264.7 Cells

Here, we investigated the inhibitory effect of 2′-*O*-GH on LPS-induced phosphorylation of IκBα and degradation of IκBα. As seen in **Figure 2A**, the phosphorylated IκBα expression and IκBα degradation was markedly increased after treatment with LPS for 15 min. However, 2′-*O*-GH treatment inhibited LPS-induced IκBα phosphorylation and blocked IκBα degradation in a concentration-dependent manner. We also found that LPS-induced phosphorylation of IKKα/b in LPS-induced RAW 264.7 macrophages were significantly suppressed by treatment with 2′-*O*-GH. Next, LPS treatment resulted in an increase in nuclear p65 level, while treatment with 2′-*O*-GH reversed the effect (**Figure 2B**). We also confirmed this result with immuno-fluorescence, where 2′-*O*-GH blocked LPS-induced nuclear translocation of p65 (**Figure 2C**). Furthermore, upregulation of luciferase activity induced by LPS was apparently suppressed by 2′-*O*-GH suggesting that 2′-*O*-GH down-regulated the expression of NF-κB-regulated genes at the transcriptional level (**Figure 2D**).

It has been known that activation of MAPKs (ERK1/2, p38, and JNK) is involved in modulating the NF-κB-regulated gene activation (Wu et al., 2017) As shown in **Figure 2E**, 2′-*O*-GH significantly suppressed the LPS-induced phosphorylation of ERK1/2, p38, and JNK in a concentration-dependent manner. Furthermore, pretreatment with MAPK inhibitors (U0126, SP600125, and SB203580) suppressed the production of NO (Supplementary Figure S1). These results indicated that 2′-*O*-GH effectively inhibited the activation of both NF-κB and MAPK in LPS-induced macrophages.

### 2′-O-GH Regulated the Inflammatory Response by SIRT1-Mediated NF-κB Signaling Pathway in LPS-Induced RAW 264.7 Cells

**FIGURE 3:**
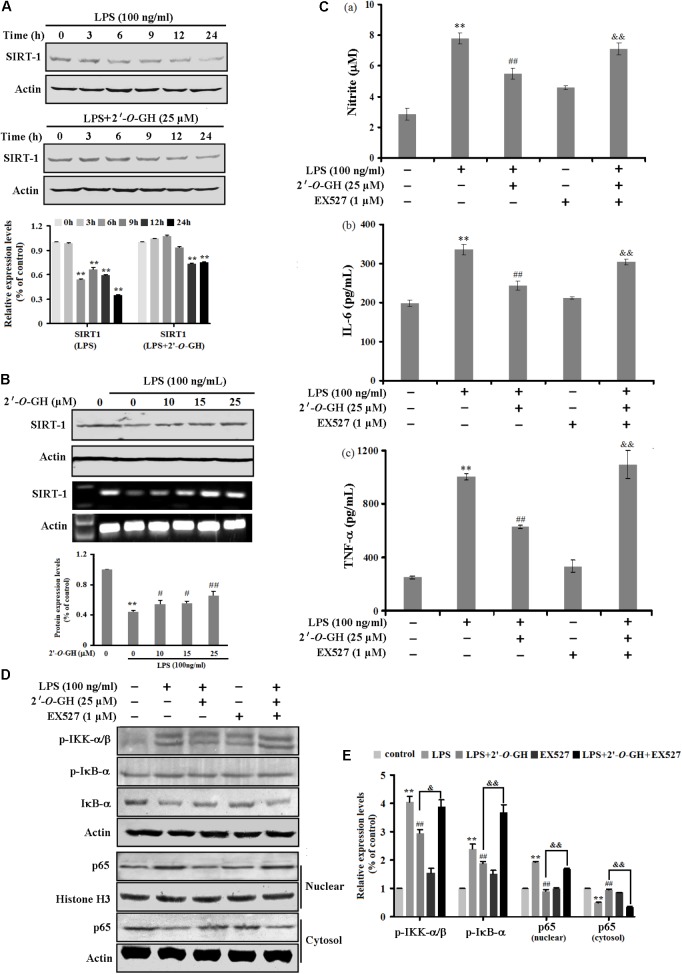
Effects of 2′-*O*-GH on LPS-stimulated SIRT1 expression in RAW264.7 cells. The cells were pretreated with 2′-*O*-GH for 2 h in the presence or absence of EX527, and subsequently incubated with 100 ng/mL LPS for the indicated time periods **(A)**, 24 h **(B–C)**, 15 min **(D)**, or 1 h **(E)**. **(A,B)** The mRNA and protein expression of SIRT1 were measured. Actin was used as an internal control. **(C)** The secretion of cytokines into culture media was evaluated by ELISA assay. **(D)** p-IκB-α, IκB-α, p-IKKα/β, and nuclear and cytosolic p65 were analyzed by Western blot analysis. Quantitative data were presented as mean ± SEM (*n* = 3). ^∗∗^*p* < 0.01 versus control, ^##^*p* < 0.01 versus LPS, ^&&^*p* < 0.01 versus 2′-*O*-GH + LPS (one-way ANOVA followed by Tukey *post hoc* multiple comparison tests).

Previous studies reveal that SIRT1 displays a pivotal role for anti-inflammatory effects in RAW 264.7 cells exposed to LPS (Zhang et al., 2017). Based on the above result, we first examined the effects of 2′-*O*-GH on the protein and mRNA expression of SIRT1 stimulated with 100 ng/mL LPS in RAW 264.7 cells. Western blot and reverse transcriptase polymerase chain reaction (RT-PCR) analysis showed that the level of SIRT1 decreased after LPS stimulation, whereas the decreased level of SIRT1 was markedly reversed in the presence of 2′-*O*-GH (**Figures 3A,B**). This result suggests that the SIRT1 pathway might be involved in the anti-inflammatory effects of 2′-*O*-GH in LPS-induced RAW264.7 cells.

To verify this whether SIRT1 was involved in the anti-inflammatory pathways activated by 2′-*O*-GH, Ex527, an inhibitor of SIRT1, was used. As shown in **Figure 3C**, the decreased levels of TNF-α, IL-6, and NO by 2′-*O*-GH were markedly abolished after Ex527 treatment (**Figure 3C**). Furthermore, the suppressive effects of 2′-*O*-GH on IKK phosphorylation, IκBα phosphorylation, and IκBα degradation were obviously reduced in LPS-simulated RAW264.7 cells, if cells were treated with EX527 (**Figure 3D**). Consistently with the above findings, blockage of SIRT1 also significantly elevated LPS-induced p65 protein levels in the nuclear fractions (**Figure 3D**). Taken together, these results indicated that attenuating LPS-induced inflammatory by 2′-*O*-GH was, at least in part, associated with activation of the SIRT1 pathway.

### 2′-O-GH Increased Nrf2-Mediated HO-1 Expression to Attenuate LPS-Stimulated Oxidative Stress and Inflammatory Response in RAW 264.7 Cells

**FIGURE 4:**
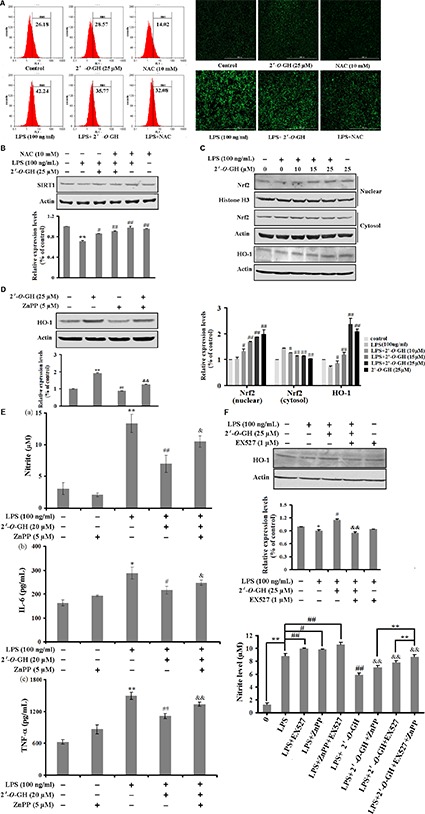
Effect of 2′-*O*-GH on the regulation of Nrf2/HO-1 pathway in LPS-induced RAW 264.7 cells. Cells were treated with indicated concentrations of 2′-*O*-GH (25 μM) in the absence or presence of NAC for 1 h, followed by stimulation with LPS (100 ng/mL) for 24 h **(A)** Flow cytometry analysis of intracellular ROS **(B)** Confocal image analysis of intracellular ROS production. **(C)** SIRT1 protein expression was determined by Western blot analysis. Cells were cultured with 100 ng/mL LPS under 2′-*O*-GH treatment in the presence or absence of 5 μM of ZnPP. **(D)** Cytoplasmic and nuclear Nrf-2 protein was detected by Western blot using corresponding antibodies. Histone H3 was used as nuclear marker and β-actin was used as cytosolic protein marker for standardization. **(E)** Level of HO-1 protein expression was determined by Western blot analysis. **(F)** The productions of Nitrite, IL-6, and TNF-α in culture supernatants were measured. The results were expressed as mean ± SD of three independent experiments. ^∗^*p* < 0.05 and ^∗∗^*p* < 0.01 compared to vehicle control, ^#^*p* < 0.05 and ^##^*p* < 0.01 versus LPS-stimulated group, ^&^*p* < 0.05 and ^&&^*p* < 0.01 versus 2′-*O*-GH + LPS group (one-way ANOVA followed by Tukey *post hoc* multiple comparison tests).

Activated macrophages release massive amounts of ROS during inflammation (Wang et al., 2016). We investigated the effects of 2′-*O*-GH on intracellular ROS levels in LPS-stimulated RAW 264.7 cells. As shown in **Figure 4A**, 2′-*O*-GH inhibited LPS-induced intracellular accumulation of ROS. Moreover, a generally accepted antioxidant, NAC, was used as positive control. NAC attenuated the LPS-induced ROS production. Similar to 2′-*O*-GH, NAC also inhibited LPS-induced secretion of NO (Supplementary Figure S2). A previous study showed a close association between oxidative stress and SIRT1, oxidative stress-mediated reduction of SIRT1 leads to the loss of its control on target proteins, thereby enhancing the inflammatory events (Kauppinen et al., 2013). Our results showed that NAC significantly blocked LPS-induced SIRT1 down-regulation in RAW 264.7 cells, whereas treatment with 10 mM NAC alone did not affect the expression of SIRT1 compared with the normal group (**Figure 4B**).

Our previous study also discovered that 2′-*O*-GH induced HO-1 expression through the Nrf2/ARE-mediated antioxidant pathway in HepG2 cells (Wang et al., 2017). Thus, we examined whether the anti-inflammatory role of 2′-*O*-GH was dependent on Nrf2 signaling. Treatment with 2′-*O*-GH showed a concentration-dependent increase in nuclear Nrf2 translocation, no matter of whether 2′-*O*-GH was treated alone or co-incubated with LPS (**Figures 4C,D**). Further studies indicated that 2′-*O*-GH also up-regulated HO-1 expression regardless of LPS. Next, ZnPP as specific HO-1 inhibitor was utilized to examine the role of HO-1 for the anti-inflammatory effects of 2′-*O*-GH. As shown in **Figure 4E**, ZnPP significantly reversed the 2′-*O*-GH-mediated suppression of NO, IL-6, and TNF-α production in LPS-stimulated RAW264.7 cells. Furthermore, we investigated the SIRT1/HO-1 signaling pathway through Western blot analysis after inhibiting SIRT1 using the inhibitor EX527. The results indicated that 2′-*O*-GH treatment significantly up-regulated HO-1 expression in the presence of LPS, whereas this effect was significantly abolished in the LPS + 2′-*O*-GH + EX527 group (**Figure 4F**). Additionally, combination of EX527 with Znpp before 2′-*O*-GH treatment significantly increased NO content compared to 2′-*O*-GH + Znpp group (**Figure 4F**).

### 2′-O-GH Enhanced the Survival Rate of LPS-Challenged Mice and Inhibited the Production of Inflammatory Cytokines in Mouse Serum

**FIGURE 5:**
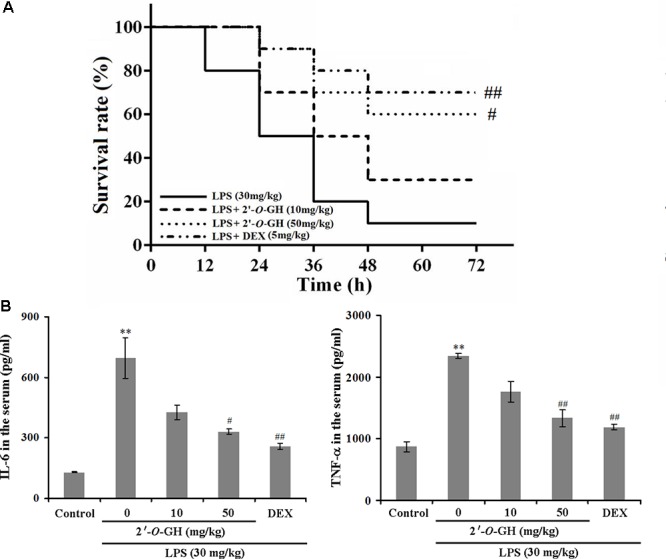
Effect of 2′-*O*-GH on production of inflammatory mediators and lethality in LPS-induced septic shock model. Mice were injected with 2′-*O*-GH (10 or 50 mg/kg, i.p.) or vehicle 12 and 1 h before LPS injection (30 mg/kg, i.p.), and the blood samples were harvested 12 h after LPS injection. **(A)** Survival rates of these mice (*n* = 10) were observed over the next 72 h. Both Log-rank test and Gehan-Breslow-Wilcoxon test was performed to evaluate overall survival rates. **(B)** The levels of TNF-α and IL-6 in the mouse serum were measured by the ELISA reaction. ^∗∗^*p* < 0.01 versus control group; ^#^*p* < 0.05, ^##^*p* < 0.01 versus the LPS group (one-way ANOVA followed by Tukey *post hoc* multiple comparison tests).

LPS is a critical factor to induce severe and systemic inflammatory responses, which represents a good model for exploring the inflammatory process *in vivo* (Li et al., 2016). As shown in **Figure 5A**, LPS administration (30 mg/kg, i.p.) resulted in 90% mortality 48 h post injection, but the survival rates in the 2′-*O*-GH (10 and 50 mg/kg) or DEX (5 mg/kg) treated groups were 30, 60, and 70%, respectively, 72 h post-injection. Since these results indicated that 2′-*O*-GH protected from LPS-induced lethality *in vivo*, we further investigated whether 2′-*O*-GH affected the production of TNF-α and IL-6 in LPS-challenged mice. As shown in **Figure 5B**, TNF-α and IL-6 in serum were significantly increased, if mice were treated with LPS alone. However, treatment with 2′-*O*-GH or DEX significantly inhibited the production of TNF-α and IL-6.

### 2′-O-GH Enhanced SIRT1 and Nrf2-Mediated Antioxidant Enzymes to Attenuate NF-κB Activation in Liver Tissue

**FIGURE 6:**
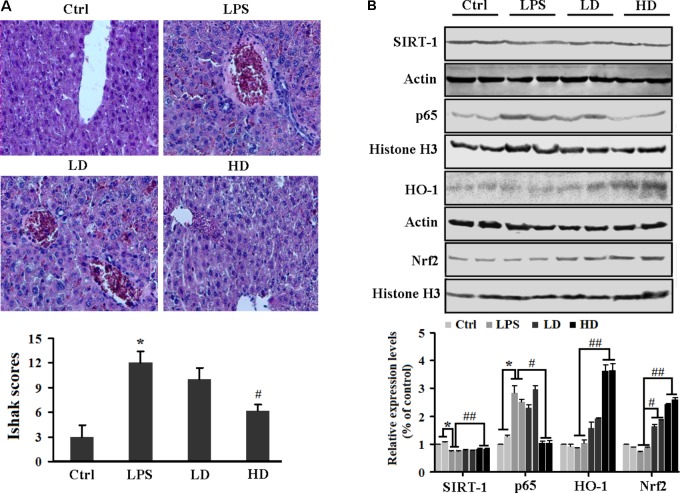
Effects of 2′-*O*-GH on the liver damage and inflammatory-related protein expression in LPS-challenged mice. Mice were injected with 2′-*O*-GH (10 or 50 mg/kg, i.p.) or vehicle 12 and 1 h before LPS injection (30 mg/kg, i.p.), and the blood samples were harvested 12 h after LPS injection. **(A)** Micrographs of renal sections stained with H&E at a magnification of 200×. **(B)** Liver protein levels of SIRT1, HO-1, nuclear Nrf-2, and p65 were detected by Western blotting (ctrl, normal control group; LD, low dosage group, 10 mg/kg 2′-*O*-GH, i.p; HD, high dosage group, 50 mg/kg 2′-*O*-GH, i.p. ^∗^*p* < 0.05, ^∗∗^*p* < 0.01 versus control group; ^#^*p* < 0.05, ^##^*p* < 0.01 versus LPS group (one-way ANOVA followed by Tukey *post hoc* multiple comparison tests).

For the liver injury, we first evaluated the serum ALT and AST levels. We found that liver damage markers (AST and ALT) were significantly increased in LPS-challenged mice. However, treatment with 2′-*O*-GH significantly reduced the activity of ALT and AST in a dose-dependent manner (**Table 1**). The histological examination did not show pathological abnormalities in the control mice. In contrast, the administration of 2′-*O*-GH suppressed LPS-induced hepatic cell, necrosis hemorrhage, and infiltration of inflammatory cells into the cavities of liver tissue (**Figure 6A**). To determine whether the mechanisms *in vitro* are also operative in endotoxic shock mice, Nrf2 and SIRT1 expressions were first detected in livers. The expressions of SIRT1 and nuclear Nrf2 were significantly increased in 2′-*O*-GH treatment groups, which in turn also increased the expression of the antioxidant enzymes HO-1, SOD, and GSH-Px (**Table 1**). We further investigated the protein expression of NF-κB p65 in tissues by Western blot analysis. As shown in **Figure 6B**, 2′-*O*-GH treatment obviously inhibited NF-κB p65 protein expression in liver, if compared with the control group. Based on these results, it was deduced that increased expression of Nrf2 and SIRT1, as well as inhibition of NF-κB activation by 2′-*O*-GH were relevant for its anti-inflammatory effects in LPS-induced endotoxin shock.

**Table 1:** The effect of 2′-O-GH on liver damage markers and activities of SOD and GSH-Px *in vivo*.

	Control	LPS	2′-*O*-GH (10 mg/kg)	2′-*O*-GH (50 mg/kg)
AST (U/L)	16.5 ± 1.32	117.4 ± 6.74^∗∗^	96.9 ± 4.71	82.5 ± 3.85^#^
ALT (U/L)	7.1 ± 0.91	54.4 ± 6.15^∗∗^	44.2 ± 1.78	34.8 ± 3.29^#^
SOD (U/mg protein)	85.8 ± 2.69	61.9 ± 1.29^∗∗^	67.4 ± 1.54	78.1 ± 1.17^##^
GSH-Px (U/mg protein)	122.4 ± 5.48	61.4 ± 5.88^∗∗^	79.8 ± 3.68	91.3 ± 6.02^#^

*The serum AST and ALT levels, and the liver SOD and GSH-Px activities were measured. These data are expressed as the mean ± SEM, n = 5. ^∗^*p* < 0.05 and ^∗∗^*p* < 0.01 versus control group, ^#^*p* < 0.05 and ^##^*p* < 0.01 versus model group of LPS-induced mice (one-way ANOVA followed by Tukey *post hoc* multiple comparison tests)*.

## Discussion

Although the therapeutic potential of *P. incarnata* Fisch. has been reported for hypertension, cardiovascular disease, gastric, and pulmonary hemorrhage (Luo et al., 2004; Sun et al., 2011), the plant’s anti-inflammatory effect remains unclear. 2*′-O*-GH, a main compound isolated from *P. incarnata* Fisch., has strong antioxidant activity and inhibited ischemia and reperfusion-induced arrhythmia (Feng et al., 2002; Wang et al., 2017). In the present study, we demonstrated for the first time the anti-inflammatory activities of 2′-*O*-GH both *in vitro* and *in vivo*. 2′-*O*-GH significantly inhibited the LPS-stimulated production of NO, TNF-α, and IL-6 in RAW 264.7 cells and mice serum. 2′-*O*-GH inhibited the LPS-induced phosphorylation of MAPKs and down-regulated the NF-κB-mediated transcriptional activity. 2′-*O*-GH also decreased intracellular ROS levels, which was associated with up-regulation of Nrf2/HO-1 axis. In addition, upregulation of SIRT1 by 2′-*O*-GH was responsible for NF-κB inhibition. Finally, 2′-*O*-GH treatment significantly reduced LPS-induced mortality and attenuated the liver injury. These results suggest that 2′-*O*-GH may have considerable potential for endotoxin shock treatment.

Inflammation is a complex processes triggered by foreign pathogens or tissue injury to eliminate harmful stimuli as well as to initiate the healing and repair process of the damaged tissue (Laveti et al., 2013). LPS is a major constituent of the cell wall of Gram-negative bacteria, which activates macrophages and induces inflammatory mediators and cytokines (Mo et al., 2014; Wu et al., 2017). Overwhelming secretion of pro-inflammatory cytokines causes severe tissue damage, multiple organ failure, or death (Ozer et al., 2017). Thus, blocking the effects of pro-inflammatory molecules (e.g., NO, TNF-α, and IL-6) offers an attractive therapeutic strategy. Our results indicated that 2′-*O*-GH significantly reduced production of these pro-inflammatory molecules in LPS-induced RAW264.7 cells. Additionally, 2′-*O*-GH dose-dependently suppressed protein expression of iNOS, TNF-α, and IL-6, which supported the above conclusion that 2′-*O*-GH attenuated LPS-induced inflammation through regulation of pro-inflammatory mediators.

Recent studies indicated that NF-κB is a pivotal transcription factor that induces the pro-inflammatory cytokine secretion and iNOS activity (Wang et al., 2016). Upon stimulation with LPS, IκB is rapidly phosphorylated by IKKα/β and subsequent degraded by proteasomes, which results in dissociation from IκB and nuclear translocation of NF-κB. In the nucleus, it binds to the promoter of target genes and activates transcription (Biswas and Bagchi, 2016). In the present study, we provided direct evidence that 2′-*O*-GH attenuated LPS-induced NF-κB activity and translocation of p65 protein to the nucleus through inhibition of IKKα/β phosphorylation and the degradation of IκBα. This mechanism is similar to the action of hyperoside (Kim et al., 2011). In addition, phosphorylation of MAPKs is part of the upstream signaling pathway for NF-κB activation in response to external stimuli (Wu et al., 2017). Furthermore, MAPKs signaling pathways play an important role in controlling inflammatory mediators in LPS-stimulated RAW 264.7 cells (Zou et al., 2018). Our results revealed that 2′-*O*-GH attenuated the phosphorylation of all three MAPK molecules, indicating that its anti-inflammatory activity was partially associated with blocking the MAPK signaling pathways.

SIRT1 plays a principal role in modulating the NF-κB-driven production of inflammatory cytokines. Xu et al. (2016) reported that SIRT1/3 activation by resveratrol attenuated AKI following sepsis in a septic rat model. Zhang et al. (2017) found that up-regulation of SIRT1 by berberine suppressed NF-κB-mediated inflammatory responses in LPS-induced RAW264.7 cells. Risitano et al. (2014) showed that a flavonoid fraction of bergamot juice reduced LPS-induced inflammatory responses through SIRT1-mediated NF-κB inhibition in THP-1 monocytes. These findings provide a rationale for the activation of SIRT1 in inflammatory diseases. In our study, 2′-*O*-GH treatment reversed both sepsis-induced down-regulation of SIRT1 expression and pro-inflammatory cytokines (TNF-α and IL-6) secretion. We also found that addition of EX527, a specific inhibitor of SIRT1, significantly alleviated the inhibitory effect of 2′-*O*-GH on NF-κB nucleocytoplasmic translocation in response to LPS, suggesting that 2′-*O*-GH-induced NF-κB inhibition may be related to the upregulation of SIRT1.

During inflammatory conditions, activated macrophages exhibit increased ROS accumulation (Wang et al., 2016). Excess amounts of ROS stimulated the expression of various inflammation-associated genes via activation of the NF-κB signaling pathway (Ko et al., 2017). HO-1 regulated by Nrf2/ARE signaling pathway played an important role in inhibiting the production of pro-inflammatory cytokines and the expression of iNOS and COX-2. Induction of Nrf2/HO-1 represents an effective cellular strategy to counteract oxidative stress and inflammatory response in activated macrophages. For example, Kang et al. (2013) reported that quercetin (2-(3,4-Dihydroxyphenyl)-3,5,7-trihydroxy-4*H*-1-benzopyran-4-one) activated Nrf2 signaling and attenuated oxidative stress and inflammation by facilitating HO-1 activity. Lv et al. (2015) reported that tenuigenin (13-(chloromethyl)-2,3-dihydroxy-4,6*a*,11,11,14b-pentamethyl-2,3,4*a*,5,6,7,8,9,10,12,12*a*,13,14,14*a*-tetradecahydro-1*H*-picene-4,8*a*-dicarboxylic acid), a natural product, inhibited production of the inflammatory mediator, which was associated with enhanced Nrf2/HO-1 expression and suppression of the NF-κB signal pathway. According to our previous studies, 2′-*O*-GH exhibited a good antioxidant effect on H_2_O_2_-stimulated HepG2 cells by decreasing ROS production and inducing HO-1 expression via the Nrf2/antioxidant response element (ARE) pathway (Wang et al., 2017). We observed that 2′-*O*-GH exerted its antioxidant activity in LPS-stimulated RAW264.7 by suppressing ROS production and enhancing HO-1 expression via Nrf2/ARE signaling.

Sepsis, a systemic inflammatory response, is a leading cause of death and failure of organ systems resulting from the inappropriate regulation of the immune system (Hung et al., 2017). LPS-induced endotoxin shock is usually considered as well-established animal model of sepsis to evaluate the anti-inflammatory effect of test compounds (Li et al., 2016). In this study, we assessed the suppressive effects of 2′-*O*-GH on LPS-induced systemic inflammation and liver injury. Exposure to high doses of LPS led to an increase of cytokines (TNF-α, IL-6) in serum, whereas treatment with 2′-*O*-GH reduced the serum levels of TNF-α and IL-6. In addition, 2′-*O*-GH also reduced mouse mortality and hemorrhage, necrosis, and infiltration of inflammatory cells in liver tissues. Therefore, 2′-*O*-GH may have an anti-inflammatory potential in endotoxic shock therapy.

## Conclusion

In conclusion, this is the first study to report that 2′-*O*-GH not only effectively inhibited inflammatory responses through a SIRT1-mediated inhibition of NF-κB but also attenuated LPS-induced ROS generation via up-regulated Nrf2/HO-1expression. In addition, 2′-*O*-GH administration significantly protected from LPS-induced liver tissue damage and increased the survival rate upon LPS-induced septic shock. These findings on the anti-inflammatory action of 2′-*O*-GH and its underlying mechanisms may be a potential therapeutic agent for inflammatory diseases.

## Author Contributions

Y-JF, PW, and CG conceived and designed the study. PW, CG, NG, S-DZ, and JZ performed the experiments. WW and L-PY analyzed the data. PW and CG wrote the paper. Y-JF and TE reviewed and edited the manuscript. All authors read and approved the manuscript.

## Conflict of Interest Statement

The authors declare that the research was conducted in the absence of any commercial or financial relationships that could be construed as a potential conflict of interest. The reviewer CF and handling Editor declared their shared affiliation.

## Abbreviations

2*′-O*-GH: 2′-*O*-galloylhyperin
ALT: alanine aminotransferase
AST: aspartate aminotransferase
DEX: dexamethasone
DMEM: Dulbecco’s Modified Eagel Medium
FBS: Fetal bovine serum
GSH: glutathione
H&E: hematoxylin and eosin
IL-6: interleukin 6
iNOS: inducible nitric oxide synthase
LPS: lipopolysaccharide
MAPK: Mitogen-activated protein kinase
NAC: N-acetyl-L-cysteine
NF-κB: nuclear factor-κB
Nrf2: nuclear transcription factor erythroid 2-related factor
ROS: reactive oxygen species
SIRT1: silent mating type information regulation 2 homolog 1
SOD: superoxide dismutase
TNF-α: tumor necrosis factor α.

## References

[B1] Biswas R. Bagchi A. (2016). NFκB pathway and inhibition: an overview. *Comput. Mol. Biol.* 6 1–20. 10.5376/cmb.2016.06.0001

[B2] Cheng X. Wang H. Yang J. Cheng Y. Wang D. Yang F. (2018). Arctigenin protects against liver injury from acute hepatitis by suppressing immune cells in mice. *Biomed. Pharmacother.* 102 464–471. 10.1016/j.biopha.2018.03.060 29579707

[B3] Chiavaroli A. Brunetti L. Orlando G. Recinella L. Ferrante C. Leone S. (2010). Resveratrol inhibits isoprostane production in young and aged rat brain. *J. Biol. Regul. Homeost. Agents* 24 441–446. 21122283

[B4] Dullius A. Goettert M. I. de Souza C. F. V. (2018). Whey protein hydrolysates as a source of bioactive peptides for functional foods – Biotechnological facilitation of industrial scale-up. *J. Funct. Foods*, 42 58–74. 10.1016/j.jff.2017.12.063 21917640

[B5] Feng W. Y. Yuan B. X. Hou J. Y. Wang J. X. (2002). Effects of 2′′-o-galloylhyperin on arrhythmias in an isolated tissue model of hypoxia and reperfusion. *Chin. Pharmacol. Bull.* 18 358–359.

[B6] Hung Y. L. Fang S. H. Wang S. C. Cheng W. C. Liu P. L. Su C. C. (2017). Corylin protects LPS-induced sepsis and attenuates LPS-induced inflammatory response. *Sci. Rep.* 7:46299 10.1038/srep46299 28397806 PMC5387730

[B7] Hwang J. W. Yao H. Caito S. Sundar I. K. Rahman I. (2013). Redox regulation of SIRT1 in inflammation and cellular senescence. *Free Radic. Biol. Med.* 61 95–110. 10.1016/j.freeradbiomed.2013.03.015 23542362 PMC3762912

[B8] Kang C. H. Choi Y. H. Moon S. K. Kim W. J. Kim G. Y. (2013). Quercetin inhibits lipopolysaccharide-induced nitric oxide production in BV2 microglial cells by suppressing the NF-κB pathway and activating the Nrf2-dependent HO-1 pathway. *Int. Immunopharmacol.* 17 808–813. 10.1016/j.intimp.2013.09.009 24076371

[B9] Kauppinen A. Suuronen T. Ojala J. Kaarniranta K. Salminen A. (2013). Antagonistic crosstalk between NF-κB and SIRT1 in the regulation of inflammation and metabolic disorders. *Cell. Signal.* 25 1939–1948. 10.1016/j.cellsig.2013.06.007 23770291

[B10] Kim S. J. Um J. Y. Hong S. H. Lee J. Y. (2011). Anti-inflammatory activity of hyperoside through the suppression of nuclear factor-κB activation in mouse peritoneal macrophages. *Am. J. Chin. Med.* 39 171–181. 10.1142/S0192415X11008737 21213407

[B11] Ko E. Y. Cho S. H. Kwon S. H. Eom C. Y. Jeong M. S. Lee W. (2017). The roles of NF-κB and ROS in regulation of pro-inflammatory mediators of inflammation induction in Lps-stimulated zebrafish embryos. *Fish Shellfish Immunol.* 68 525–529. 10.1016/j.fsi.2017.07.041 28743626

[B12] Laveti D. Kumar M. Hemalatha R. Sistla R. Naidu V. G. Talla V. (2013). Anti-inflammatory treatments for chronic diseases: a review. *Inflamm. Allergy Drug Targets* 12 349–361. 10.2174/18715281113129990053 23876224

[B13] Li K. K. Shen S. S. Deng X. Shiu H. T. Siu W. S. Leung P. C. (2018). Dihydrofisetin exerts its anti-inflammatory effects associated with suppressing Erk/p38 Mapk and Heme Oxygenase-1 activation in lipopolysaccharide-stimulated Raw 264.7 macrophages and carrageenan-induced mice paw edema. *Int. Immunopharmacol.* 54 366–374. 10.1016/j.intimp.2017.11.034 29202300

[B14] Li T. Zhang J. Feng J. Li Q. Wu L. Ye Q. (2013). Resveratrol reduces acute lung injury in a LPS-induced sepsis mouse model via activation of Sirt1. *Mol. Med. Rep.* 7 1889–1895. 10.3892/mmr.2013.1444 23625030

[B15] Li W. Zhang H. Niu X. Wang X. Wang Y. He Z. (2016). Effects and mechanisms of cavidine protecting mice against LPS-induced endotoxic shock. *Toxicol. Appl. Pharmacol.* 305 46–54. 10.1016/j.taap.2016.05.021 27260672

[B16] Luo D. Q. Yang Y. Z. Song L. Wang J. X. (2004). Advances in studies on special plants of *Pyrola* L. in China. *Chin. Tradit. Herb. Drugs* 35 463–465.

[B17] Lv H. Zhu C. Liao Y. Gao Y. Lu G. Zhong W. (2015). Tenuigenin ameliorates acute lung injury by inhibiting NF-κB and MAPK signalling pathways. *Respir. Physiol. Neurobiol.* 216 43–51. 10.1016/j.resp.2015.04.010 25930113

[B18] Menon D. Coll R. Board P. G. (2014). Glutathione transferase omega 1 is required for the lipopolysaccharide-stimulated induction of NADPH oxidase 1 and the production of reactive oxygen species in macrophages. *Free Radic. Biol. Med.* 73 318–327. 10.1016/j.freeradbiomed.2014.05.020 24873723

[B19] Mo C. Wang L. Zhang J. Numazawa S. Tang H. Tang X. (2014). The crosstalk between Nrf2 and AMPK signal pathways is important for the anti-inflammatory effect of berberine in LPS-stimulated macrophages and endotoxin-shocked mice. *Antioxid. Redox Signal.* 20 574–588. 10.1089/ars.2012.5116 23875776 PMC3901384

[B20] Ozer E. K. Goktas M. T. Kilinc I. Bariskaner H. Ugurluoglu C. Iskit A. B. (2017). Celecoxib administration reduced mortality, mesenteric hypoperfusion, aortic dysfunction and multiple organ injury in septic rats. *Biomed. Pharmacother.* 86 583–589. 10.1016/j.biopha.2016.11.102 28024294

[B21] Risitano R. Currò M. Cirmi S. Ferlazzo N. Campiglia P. Caccamo D. (2014). Flavonoid fraction of Bergamot juice reduces LPS-induced inflammatory response through SIRT1-mediated NF-κB inhibition in THP-1 monocytes. *PLoS One* 9 e107431. 10.1371/journal.pone.0107431 25260046 PMC4178028

[B22] Sun C. Y. Chen Z. Wang W. W. Li Y. Dong Y. L. Wang L. J. (2011). Research on the development and utilization of *Pyrola* resources. *North. Hortic.* 1 220–222.

[B23] Wang P. Gao Y. M. Sun X. Guo N. Li J. Wang W. (2017). Hepatoprotective effect of 2′-O-galloylhyperin against oxidative stress-induced liver damage through induction of Nrf2/ARE-mediated antioxidant pathway. *Food Chem. Toxicol.* 102 129–142. 10.1016/j.fct.2017.02.016 28213291

[B24] Wang P. Qiao Q. Li J. Wang W. Yao L. P. Fu Y. J. (2016). Inhibitory effects of geraniin on LPS-induced inflammation via regulating NF-κB and Nrf2 pathways in RAW 264.7 cells. *Chem. Biol. Interact.* 253 134–142. 10.1016/j.cbi.2016.05.014 27181634

[B25] Wu L. Fan Y. Fan C. Yu Y. Sun L. Jin Y. (2017). Licocoumarone isolated from *Glycyrrhiza uralensis* selectively alters LPS-induced inflammatory responses in RAW 264.7 macrophages. *Eur. J. Pharmacol.* 801 46–53. 10.1016/j.ejphar.2017.02.049 28263754

[B26] Xu S. Gao Y. Zhang Q. Wei S. Chen Z. Dai X. (2016). SIRT1/3 activation by resveratrol attenuates acute kidney injury in a septic rat model. *Oxid. Med. Cell. Longev.* 2016:7296092. 10.1155/2016/7296092 28003866 PMC5149703

[B27] Yao X. H. Zhang D. Y. Luo M. Jin S. Zu Y. G. Efferth T. (2015). Negative pressure cavitation-microwave assisted preparation of extract of *Pyrola incarnata* Fisch. rich in hyperin, 2′-O-galloylhyperin and chimaphilin and evaluation of its antioxidant activity. *Food Chem.* 169 270–276. 10.1016/j.foodchem.2014.07.115 25236226

[B28] Yao X. H. Zhang D. Y. Zu Y. G. Fu Y. J. Luo M. Gu C. B. (2013). Free radical scavenging capability, antioxidant activity and chemical constituents of *Pyrola incarnata* Fisch. leaves. *Ind. Crops Prod.* 49 247–255. 10.1016/j.indcrop.2013.04.058

[B29] Zhang H. Shan Y. Wu Y. Xu C. Yu X. Zhao J. (2017). Berberine suppresses Lps-induced inflammation through modulating Sirt1/nf-κb signaling pathway in Raw 264.7 cells. *Int. Immunopharmacol.* 52 93–100. 10.1016/j.intimp.2017.08.032 28888780

[B30] Zhang Q. J. Wang Z. Chen H. Z. Zhou S. Zheng W. Liu G. (2008). Endothelium-specific overexpression of class III deacetylase SIRT1 decreases atherosclerosis in apolipoprotein E-deficient mice. *Cardiovasc. Res.* 80 191–199. 10.1093/cvr/cvn224 18689793 PMC3657473

[B31] Zou Y. H. Zhao L. Xu Y. K. Bao J. M. Liu X. Zhang J. S. (2018). Anti-inflammatory sesquiterpenoids from the Traditional Chinese Medicine *Salvia plebeia*: regulates pro-inflammatory mediators through inhibition of NF-κκB and Erk1/2 signaling pathways in LPS-induced Raw 264.7 cells. *J. Ethnopharmacol.* 210 95–106. 10.1016/j.jep.2017.08.034 28847754

