# Supplementary material for: 2′-O-Galloylhyperin Isolated From Pyrola incarnata Fisch. Attenuates LPS-Induced Inflammatory Response by Activation of SIRT1/Nrf2 and Inhibition of the NF-κB Pathways in Vitro and Vivo
Source: Front Pharmacol. 2018 Jun 27;9:679. doi: 10.3389/fphar.2018.00679 (PMC6030669; doi:10.3389/fphar.2018.00679)
Supplement: Supplementary file 1 [file Presentation_1.ZIP › SUPPLEMENTARY INFORMATION.doc]

SUPPLEMENTARY INFORMATION:

Fig. S1

**Fig. S1. Effect of MAPK inhibitors on LPS-induced NO production.**

Cells were pretreatment with inhibitors, then stimulated with LPS (100 ng/ml) for 24 h. Nitrite production was measured by Griess reagents. Each bar represents the mean ± SD of three independent experiments. **p < 0.01 compared to vehicle control, ^#^p < 0.05 and ^##^p < 0.01 versus LPS-stimulated groups.

Fig. S2

**Fig. S2.** **Effect of NAC on** **LPS-induced NO production.**

Cells were pretreated with NAC (10 mM) for 1 h, and then stimulated with LPS (100 ng/mL) for 24 h. Nitrite production was measured by Griess reagents. Each bar represents the mean ± SD of three independent experiments. ***p < 0.05 and ****p < 0.01 vs control, *#*p < 0.05 and *##*p < 0.01 versus JG treatment.
